# A failure of Fourier transform infrared spectroscopy to type *Burkholderia* isolates from chronically infected patients with cystic fibrosis

**DOI:** 10.1128/spectrum.02214-23

**Published:** 2023-10-04

**Authors:** Jan Tkadlec, Jana Prasilova, Pavel Drevinek

**Affiliations:** 1 Department of Medical Microbiology, Charles University and Motol University Hospital, Prague, Czechia; University of Arizona/Banner HealthPathology, Tucson, Arizona, USA

**Keywords:** cystic fibrosis, *Burkholderia*, FT-IR, epidemiology

## LETTER

We read with great interest the paper by Barker et al. (1) presenting the application of Fourier transform infrared spectroscopy (FT-IR) for typing of *Burkholderia cenocepacia* epidemic clone ET12. In the past, our cystic fibrosis (CF) center faced a large outbreak caused by another *Burkholderia cenocepacia* global epidemic lineage, termed ST-32 (2, 3). We developed a complex surveillance program to prevent its further spread, which combined a nested PCR-based detection from respiratory samples, strain-specific PCR, and random amplified polymorphic DNA and/or multilocus sequence typing (MLST). In reference to the study of Barker et al., we aimed to check whether FT-IR can discriminate chronic infection with the same *Burkholderia* strain from reinfection and thus replace more expensive and time-consuming MLST in the analysis workflow. Although the authors considered the results of FT-IR typing suboptimal (10 of 51 isolates were not correctly identified as ET12 or non-ET12), we tested a newer version of the platform with an increased spectral range of FT-IR. Also, as the technology was successfully adopted for typing of pathogens that produce capsular polysaccharides (*Streptococcus pneumoniae* or *Klebsiella pneumoniae*) (4 – 6), we sought the effect of the mucoid phenotype of *Burkholderia* isolates (7) on FT-IR discriminatory power.

We examined 35 clinical isolates from 16 CF patients representing 9 different sequence types (STs) of *B. cenocepacia* and 2 STs of *Burkholderia contaminans* (another clinically relevant member of the *Burkholderia cepacia* complex; Table 1). Isolates were grown on tryptic soy agar for 30 h at 37°C and processed according to the manufacturer’s instructions. Spectra of wavenumber ranging from 1,300/cm to 400/cm were acquired and analyzed using IR-Biotyper version 3.1 (Bruker Daltonics, Germany). For each isolate, an average spectrum was created from three technical replicates. Spectra were analyzed using hierarchical clustering analysis (HCA) with a 0.200 cut-off value applied.

**TABLE 1:** Isolates used in the study

Patient ID	Isolates				Isolation year	ST
1	1a				2002	ST-32
2	2a				2012	ST-32
3	3a				2011	ST-32
4	4a				2021	ST-184
5	5a				2020	ST-241
6	6a				2021	ST-482 (*B. contaminans*)
7	7a				2011	ST-628
8	8a	8b			2018–2019	ST-643 and ST-1547
9	9a				2021	ST-761
10	10a	10b	10c		2003–2010	All ST-32
11	11a	11b	11c		2002–2010	All ST-32
12	12a	12b	12c	12d	2016–2021	All ST-32
13	13a	13b	13c	13d	2016–2021	All ST-32
14	14a	14b	14c	14d	2015–2021	All ST-102 (*B. contaminans*)
15	15a	15b	15c	15d	2016–2021	All ST-234
16	16a	16b	16c		2016–2019	All ST-834

**Fig 1:**
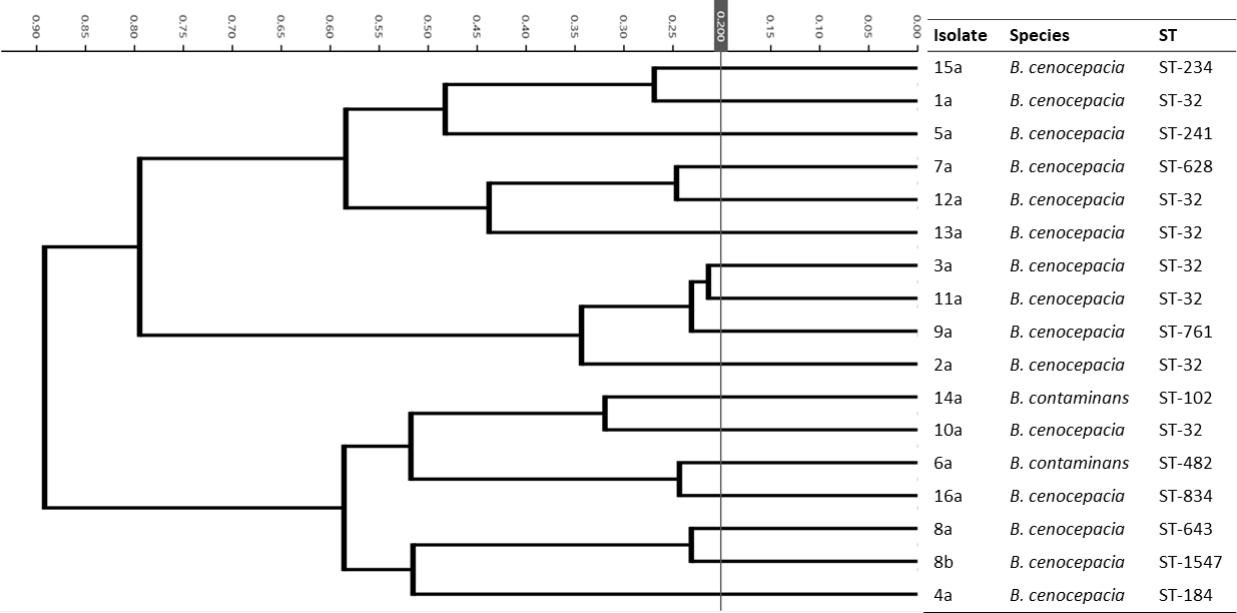
HCA differentiation by STs. Dendrogram of 17 Bcc isolates from 16 individual patients representing 11 STs.

**Fig 2:**
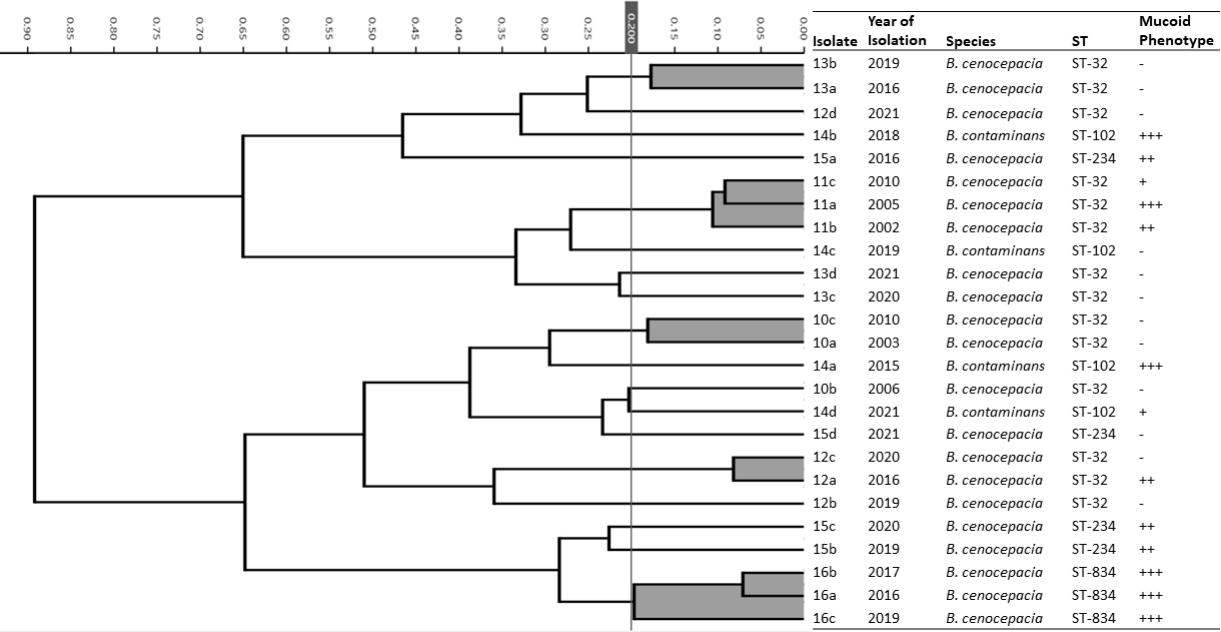
HCA differentiation by patient. Dendrogram of 25 longitudinal Bcc isolates from chronically infected CF patients. The mucoid phenotype was classified by intensity to: −, non-mucoid; +, mucoid-shiny colonies; ++, mucoid; +++, mucoid-dripping, as described previously (7).

First, we tested 17 single-patient isolates (15 *B. cenocepacia* representing 9 STs and 2 *B. contaminans* representing 2 STs), yet no clusters, matching STs or at least *Burkholderia* species, were found (Fig. 1). Second, we analyzed the spectra of 25 isolates from 7 chronically infected CF patients, obtained during a mean period of 6 yr (range 3–8 yr) of chronic infection (Table 1; patients 10 to 16). Isolates of 5 of 7 patients (12 of 25) were correctly clustered (Fig. 2), but only 2 of 5 clusters were complete and contained all isolates of respective patients (patients 11 and 16). We can speculate that mucoid phenotype attributed to their close spectra similarity, but this observation was not universal, and isolates of the other two patients (14 and 15) who also harbored mucoid isolates did not form patient-matching clusters.

In summary, we noted high variability of isolates spectra that prevented assigning isolates to known ST. Moreover, the FT-IR failed to reliably determine whether a chronic patient harbored the same *Burkholderia* strain over the course of infection. As such, we conclude that the method is not suitable for *Burkholderia* strain typing.
